# Development and Validation of a UPLC-MS/MS and UPLC-HR-MS Method for the Determination of Fumonisin B1 and Its Hydrolysed Metabolites and Fumonisin B2 in Broiler Chicken Plasma

**DOI:** 10.3390/toxins10020062

**Published:** 2018-01-31

**Authors:** Siegrid De Baere, Siska Croubels, Barbara Novak, Gerlinde Bichl, Gunther Antonissen

**Affiliations:** 1 Department of Pharmacology, Toxicology and Biochemistry, Salisburylaan 133, 9820 Merelbeke, Belgium; Siska.Croubels@ugent.be (S.C.); Gunther.Antonissen@ugent.be (G.A.); 2 BIOMIN Research Center, Technopark 1, 3430 Tulln, Austria; barbara.novak@biomin.net (B.N.); gerlinde.bichl@biomin.net (G.B.); 3 Department of Pathology, Bacteriology and Avian Diseases, Salisburylaan 133, 9820 Merelbeke, Belgium

**Keywords:** fumonisin B1, hydrolysed FB1, fumonisin B2, UPLC-MS/MS, UPLC-HR-MS, plasma, broiler chickens, toxicokinetics, Development of an UPLC-MS/MS and UPLC-HR-MS method that can be applied to investigate the toxicokinetics of Fumonisin B1 and its hydrolysed metabolites, and Fumonisin B2 in chicken plasma.

## Abstract

A sensitive and specific method for the quantitative determination of Fumonisin B1 (FB1), its partially hydrolysed metabolites pHFB1a+b and hydrolysed metabolite HFB1, and Fumonisin B2 (FB2) in broiler chicken plasma using ultra-performance liquid chromatography combined with tandem mass spectrometry (UPLC-MS/MS) was developed. The sample preparation was rapid, straightforward and consisted of a deproteinization and phospholipid removal step using an Oasis^®^ Ostro^TM^ 96-well plate. Chromatography was performed on an Acquity HSS-T3 column, using 0.3% formic acid and 10 mM ammonium formate in water, and acetonitrile as mobile phases. The MS/MS instrument was operated in the positive electrospray ionization mode and the two multiple reaction monitoring transitions were monitored for each component for quantification and identification, respectively. The method was validated in-house: matrix-matched calibration graphs were prepared and good linearity (r ≥ 0.99) was achieved over the concentration ranges tested (1–500 ng/mL for FB1 and FB2; 0.86–860 ng/mL for pHFB1a; 0.72–1430 ng/mL for pHFB1b and 2.5–2500 ng/mL for HFB1). Limits of quantification (LOQ) and detection (LOD) in plasma ranged between 0.72 to 2.5 ng/mL and 0.03 to 0.17 ng/mL, respectively. The results for the within-day and between-day precision and accuracy fell within the specified ranges. Moreover, the method was transferred to an UPLC high-resolution mass spectrometry (HR-MS) instrument in order to determine potential metabolites of HFB1, such as N-acyl-HFB1s and phase II metabolites. The method has been successfully applied to investigate the toxicokinetics and biotransformation of HFB1 in broiler chickens.

## 1. Introduction

Fumonisins (FBs) are secondary metabolites of fungi, such as *Fusarium verticillioides, Fusarium proliferatum* and *Aspergillus niger*, which frequently contaminate maize and maize-based products [1,2,3,4]. Fumonisin B1 (FB1) is the most prevalent component of the family and is hepato- and nephrotoxic in a variety of animal species, although it is poorly absorbed and rapidly excreted [1,4,5,6,7]. FB1 is associated with a number of mycotoxicoses, such as equine leukoencephalomalacia [5], porcine pulmonary oedema [5,6] and hepatocarcinoma in rats [8,9].

**Figure 1:**
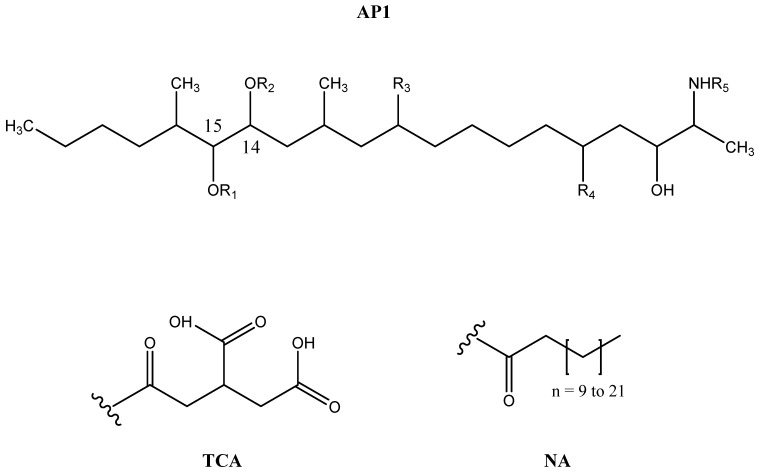
Structures of Fumonisin B1 (FB1), Fumonisin B2 (FB2), Fumonisin B3 (FB3), partially hydrolysed FB1a (pHFB1a), partially hydrolysed FB1b (pHFB1b), hydrolysed Fumonisin B1 (HFB1), N-Acyl-derivatives of HFB1 (NAHFB1) with NA-chains ranging from 12 to 24 carbon atoms; AP1 = aminopolyol backbone; TCA = tricarballylic acid side chain; NA = N-Acyl side chain [5].

FB1 (Figure 1) includes a long-chain aminopolyol (AP1) backbone (2-amino-12,16-dimethyl-3,5,10,14,15-pentahydroxyeicosane) with two ester-linked tricarballylic acids at C14 and C15 (propane-1,2,3-tricarboxylic acid, TCA) [1,10]. It is a competitive inhibitor of the enzyme ceramide synthase (CerS) and disrupts sphingolipid metabolism due to the structural similarity between FB1 and sphinganine (Sa) or sphingosine (So), which are CerS substrates [1,2,4,9]. The elevation of the free Sa/So ratio in biological matrices was suggested as an efficient biomarker.

FB1 can be converted to partially hydrolysed FB1 (pHFB1a and pHFB1b) and hydrolysed FB1 (HFB1) by the cleavage of the TCA side chains at C14 and C15 (Figure 1) through alkaline hydrolysis during food and feed processing (nixtamalization) [2,6,11]. Hence, animals can be exposed not only to the parent compound FB1, but also to the hydrolysed forms. Moreover, it has been demonstrated in pigs that FB1 can be metabolized by carboxylesterase(s) into pHFB1 and HFB1 by the intestinal microbiota and the liver [1,12], whereas it is not known if these metabolites are formed in avian species.

There is a conflicting evidence concerning the in vivo toxicity of pHFB1 and HFB1 [1,5,6,7,10]. Hahn et al. [2] investigated the occurrence and toxicity of FB1 and its (partially) hydrolysed metabolites in vivo after oral administration to rats. FB1 and its metabolites could be determined in faeces, but in urine only FB1 could be observed. Based on urinary Sa/So ratios, they supposed that both pHFB1 and HFB1 were significantly less toxic than FB1. HFB1 is not only a 10 times weaker inhibitor, but also a substrate for CerS in rat liver microsomes [2,4,5,9,13] and can be acetylated at the primary amino group with fatty acids of various chain lengths to form ceramide analogues known as N-acyl-HFB1s (NAHFB1s). In addition, some authors reported the in vivo formation of NAHFB1s in liver and kidney of rats exposed to HFB1 [5]. Therefore, elimination of the TCA side chains of FB1 by carboxylesterase is not sufficient for FB1 detoxification and elimination of the C2-amino group of HFB1 by aminotransferase is essential as well [9]. No data on the in vivo toxicokinetics of HFB1 are available in other animal species besides the rat, nor on the formation of NAHFB1s.

To extend the current knowledge of the biotransformation of FB1 and its in vivo metabolites and to investigate the toxicokinetics of these components, sensitive analytical methods are necessary to detect the generally low concentrations in biological matrices. Currently, there are only a few methods available for the determination of FB1 and its metabolites in biological matrices of animal origin: high-performance liquid chromatography (HPLC) methods with fluorescence (FL) detection after derivatisation with *o*-phthaldialdehyde (OPA) reagent were reported for the analysis of FB1 in rat plasma and urine [14] or FB1 and its metabolites in ruminal fluid [8]. To omit the time-consuming derivatisation procedure, liquid chromatography in combination with mass spectrometric detection (LC-MS) was used for the determination of FB1 and its hydrolysed or N-acetylated metabolites in animal biological matrices, such as urine, faeces and tissues [2,7,10,12] or plasma [15]. Sample preparation consisted of protein precipitation using acetonitrile (ACN) [15] or liquid-liquid extraction (LLE) in polar solvents, such as mixtures of methanol, ACN, ethyl acetate (EtOAc), chloroform or aceton with water [2,4,5,12]. In some cases, acids were added, such as hydrochloric acid, sulfuric acid, formic acid (FA) to enhance the extraction of analytes due to their acidic properties [16]. Other authors applied solid-phase extraction (SPE) [17] or a combination of protein precipitation, LLE and SPE to purify the samples prior to LC-MS analysis [7,10,18].

Until now, no data on the toxicokinetics of FB1 and its hydrolysed metabolites in avian species are available. In addition, sensitive and straightforward analytical methods for the determination of these components in animal plasma are scarce. Therefore, it was the aim of this study to develop and validate an ultra-performance liquid chromatography tandem mass spectrometry (UPLC-MS/MS) method for the simultaneous quantitative determination of FB1 and its hydrolysed metabolites (pHFB1a+b, HFB1) in broiler chicken plasma. The validated method was applied to the analysis of plasma samples that were taken as a part of a toxicokinetic (TK) study with HFB1 in chickens fed a control and a FBs contaminated diet for two weeks. To investigate possible N-acyl-metabolites of HFB1, the method was also transferred to an UPLC high-resolution mass spectrometry (HR-MS) instrument.

## 2. Results and Discussion

During method development, the following points of interest were taken into account. First, the sample preparation procedure had to be straightforward and cost-effective, since it was the final aim to analyse a high number of samples per day (*n* ≥ 96). Secondly, to be able to analyse all these samples within 24 h, the UPLC-MS/MS analysis had to be accomplished within a short run-time (≤10 min).

### 2.1. Sample Preparation

The preferred extraction methods for FBs in biological matrices (urine, faeces, tissues) are single-step or multiple-step LLE alone [2,4,5,12], or in combination with a SPE clean-up [7,10,17]. For the present study, LLE using diethylether and EtOAc at two different pH levels (2 and 7) was evaluated.

In addition, a more generic sample preparation procedure, based on protein precipitation by 1% FA in ACN in combination with phospholipid (PL) removal, using Ostro^TM^ 96-well plates was tested. This procedure was based on that reported by De Baere et al. [19] for the analysis of gamithromycin in chicken plasma. Indeed, during this study analytical problems were observed when a simple protein precipitation step with ACN was applied for sample clean-up, which could be attributed to the presence of high amounts of PLs in the deproteinized sample. Ferlazzo et al. [20] indeed reported that phosphatidylcholine and sphingomyelin levels were significantly higher in chicken plasma compared to other species tested (pigs, cows, horses and ostriches) and Chambers et al. [21] mentioned that matrix components and endogenous PLs in particular, can be a serious source of imprecision in quantitative LC-MS/MS analysis.

**Figure 2:**
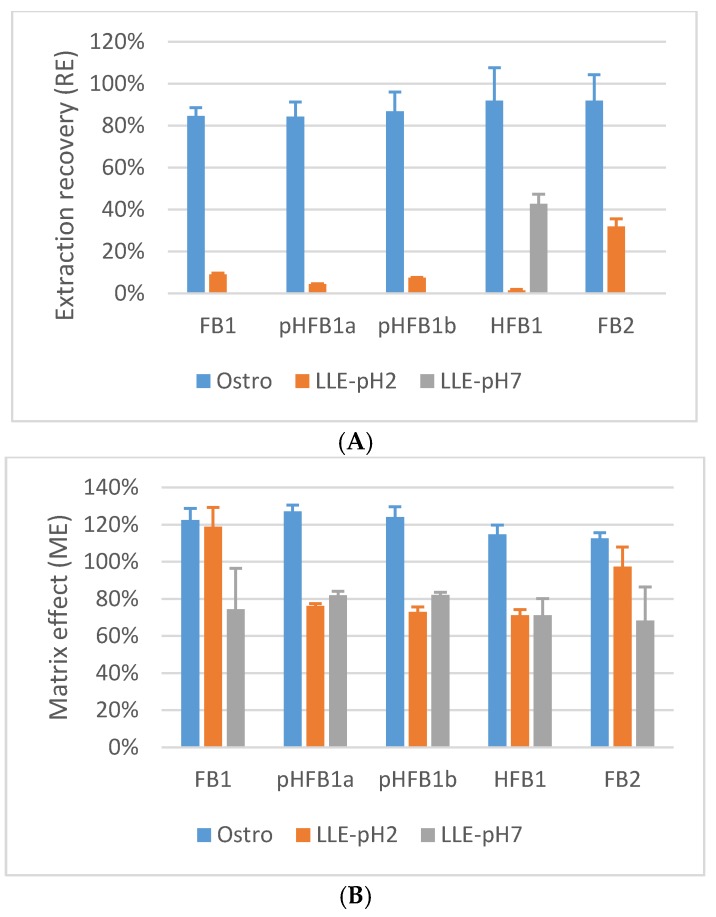

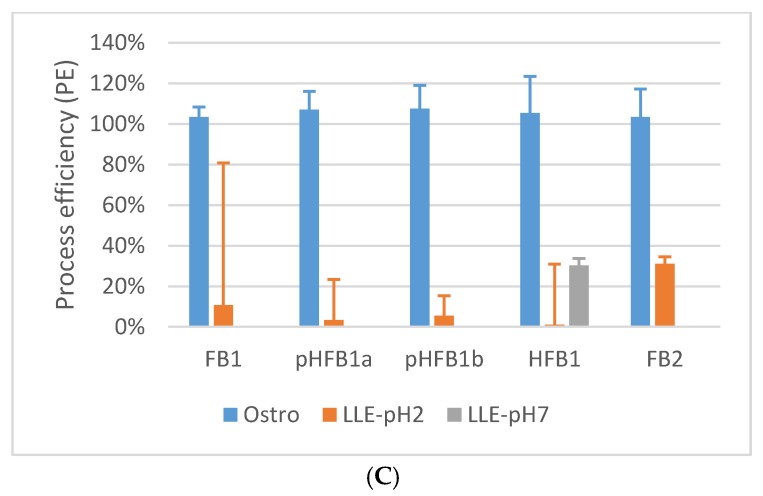
Extraction recovery (R_E_, panel **A**), matrix effect (M_E_, panel **B**) and process efficiency (P_E_, panel **C**) for FB1, FB2, pHFB1a, pHFB1b and HFB1 in chicken plasma after protein precipitation and PL removal using Ostro^TM^ 96-well plates (Ostro) and LLE using EtOAc at pH 2 (LLE-pH2) and pH 7 (LLE-pH7). Results are the mean of three replicates (*n* = 3). The analytes were spiked at the following concentration levels: 250 ng/mL (FB1 and FB2), 430 ng/mL (pHFB1a), 715 ng/mL (pHFB1b) and 1250 ng/mL (HFB1).

As can be seen in Figure 2, the best results for extraction recovery (R_E_) were obtained using the Ostro^TM^ 96-well plate (ranging between 61.6–70.8%). FB1, FB2 and the hydrolysed metabolites of FB1 could not be extracted at both pH 2 and pH 7 using diethylether as extraction solvent. Using EtOAc, low amounts of the analytes could be extracted at pH 2 (R_E_ ranging between 1.4–32.0%), whereas at pH 7 only HFB1 could be extracted with a R_E_ of 42.6%. The higher R_E_ for FB1 and its metabolites using the Ostro^TM^ 96-well plate can be explained by the chemical properties of these components. FB1 is an amphiphatic zwitterion, which is soluble in polar solvents, such as methanol or ACN. Moreover, extraction of FB1 and pHFB1a+b is enhanced at acidic pH due to the presence of two or one carboxylic acid groups, respectively, that are unionized in an acidic medium; HFB1 lacks these carboxylate groups, which results in a higher R_E_ after extraction at a neutral pH (see Figure 2A) [10]. The low R_E_ percentages for the analytes of interest after LLE could also partially be explained by the co-extraction of endogenous components, such as PLs. Phosphatidylcholine containing PLs consist of both a polar zwitterionic head group and one or two long alkyl chain(s), which makes them extremely hydrophobic [21]. This means that during LLE, PLs can be extracted from the plasma matrix with organic solvents at different pH-values due to the presence of both the hydrophobic tail and the polar zwitterionic head. Because PLs are present at high concentrations in plasma (mg/mL range) [20], compared to the concentrations of the analytes (ng/mL range), this co-extraction could have a negative impact on the R_E_ of the analytes of interest.

Since the best results for R_E_ were obtained using the Ostro^TM^ 96-well plate, this procedure was selected for the sample clean-up of FB1 and its hydrolysed metabolites in chicken plasma. In addition, this sample preparation procedure was straightforward, because protein precipitation and PL removal could be performed in one action and because there was no need for a time-consuming evaporation step or a final filtration step of the extract through a 0.22 µm filter prior to UPLC-MS/MS analysis. According to Gazotti et al. [7], solvent evaporation to complete dryness can lead to a great variability in recovery, especially at low concentrations.

Using the Ostro^TM^ 96-well plate, it was possible to extract 96 plasma samples within about 1 h, which is an advantage if a large number of samples have to be analysed as part of TK studies.

### 2.2. Liquid Chromatography

The aim of this study was to develop an UPLC-MS/MS method that could separate the analytes of interest within a maximal run-time of 10 min, since a large amount of samples (*n* ≥ 96) had to be analysed in a 24-h period. Special attention was paid to the separation of the isobaric compounds pHFB1a/pHFB1b and FB2/FB3 (see Table 1).

In the literature, chromatography of FBs and their hydrolysed metabolites was generally performed using reverse-phase C18 [7,11,15,22] or C8 [9] columns. Han et al. [16] used an Acquity UPLC HSS-T3 column (100 × 2.1 mm, dp: 1.8 µm) for the separation of FB1, FB2 and FB3 in Chinese medicines. Mobile phases consisted of water to which additives were added, such as FA [7,22], FA in combination with ammonium formate (NH_4_FA) [9] or acetic acid [15]. The organic phases consisted of acetonitrile, methanol or a combination of both, to which the same additives were added as for the aqueous phases. Gradient elution was generally applied.

For the present study, two reversed phase UPLC columns (Acquity BEH C18, 50 × 2.1 mm i.d., dp: 1.8 µm, Waters; Hypersil Gold, 50 × 2.1 mm i.d., dp: 1.9 µm, ThermoScientific) and an Acquity HSS-T3 column (100 × 2.1 mm, dp: 1.8 µm, Waters) were tested in combination with the mobile phases reported in the literature. The best results were obtained using an Acquity HSS-T3 column using water containing 0.3% FA + 10 mM NH_4_FA and acetonitrile as mobile phase A and B, respectively. A gradient elution was performed, resulting in a total run-time of 10 min. This was much shorter than other methods reported in the literature, i.e., De Girolamo et al.: 40 min [11]; Gazotti et al.: 17 min [7]; Heinl et al.: 20 min [9]; Meyer et al.: 20 min [22]. Han et al. analysed FB1, FB2 and FB3 within 5 min, but this method did not include the hydrolysed metabolites [16].

**Figure 3:**
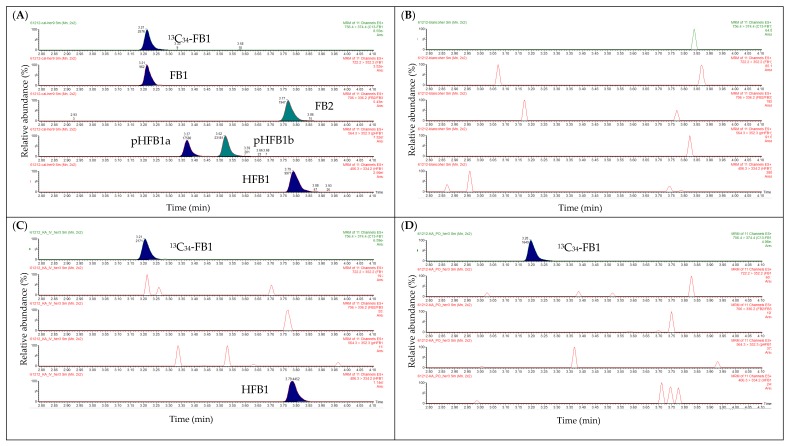
UPLC-MS/MS chromatograms of (**A**) a blank broiler chicken plasma sample spiked with FB1 (50 ng/mL), FB2 (50 ng/mL), pHFB1a (86 ng/mL), pHFB1b (143 ng/mL) and HFB1 (250 ng/mL); (**B**) a blank broiler chicken plasma sample; (**C**) a broiler chicken plasma sample that was taken 10 min after the IV administration of HFB1 (dose: 1.25 mg/kg BW), HFB1 concentration: 130.9 ng/mL; (**D**) a broiler chicken plasma sample that was taken 10 min after the oral administration of HFB1 (dose: 1.25 mg/kg BW), HFB1 concentration: not detected.

As can be seen from Figure 3A, a good separation was obtained between pHFB1a/pHFB1b. No pure analytical standards of pHFB1a and pHFB1b were available, which makes a 100% identification of these analytes difficult. The assignment of the peaks of pHFB1a/b was based on the manuscript of Heinl et al. [9]. In addition, the mean (±SD) peak area ratio of pHFB1a/b in the calibrator samples was 0.76 ± 0.06 (*n* = 30). This is in the same range as the theoretical concentration ratio of pHFB1a/b (0.57) in the standard mix, which supports the assumption that the peaks at Tr = 3.42 min and 3.56 min correspond with pHFB1a and pHFB1b, respectively.

The isobaric components FB3 (Tr = 3.59 min) and FB2 (Tr = 3.84 min) were also baseline separated (results not shown). No peaks were observed at the elution zone of the analytes of interest in a blank chicken plasma sample (Figure 3B), indicating the good specificity of the UPLC-MS/MS method.

### 2.3. Mass Spectrometry

The FBs were detected in the positive electrospray ionization (ESI) multiple reaction monitoring (MRM) mode. For each precursor ion, the two most abundant product ions were monitored and used for quantification and identification purposes, respectively (Table 1). The product ions for FB1, FB2, FB3 and pHFB1a+b were in accordance with other studies reported in the literature and consisted of the subsequent losses of the TCA side chain(s) and water, whereas the product ions for HFB1 originated from the subsequent losses of water [7,15,16].

### 2.4. High-Resolution Mass Spectrometry

HFB1 is considered as 10-fold less potent than FB1 as an inhibitor of CerS; however, it can also be a substrate for CerS in rat liver microsomes, resulting in the acetylation of HFB1 at the primary amino-group with fatty acids of various chain lengths to form ceramide analogues known as NAHFB1s [1,2,3,4,13]. NAHFB1s, which are known to be potent CerS inhibitors, were found in blood and tissues after in vivo intraperitoneal administration of HFB1 to rats (dose: low: 253 µg/kg BW; medium: 558 µg/kg BW; high: 1120 µg/kg BW). The most prevalent analogues in the liver were C24:0, C24:1, C20:0 and C22:0-NAHFB1 [5].

In order to investigate the eventual conversion of HFB1 to NAHFB1-metabolites in broiler chickens, the plasma sample extracts were injected onto an UPLC-HR-MS instrument, since no analytical standards were available of the N-acyl-metabolites. The same chromatographic conditions were used as for the HFB1 analysis. A targeted approach was used, which means that the theoretically exact masses that were calculated on the basis of the chemical formulas of the NAHFB1s were added to the processing method (see Table 2). The following NAHFB1 derivatives were searched for: C16:0, C18:0, C20:0, C22:0, C24:0 and C26:0; in addition, the presence of the unsaturated derivatives C24:1 and C26:1 was investigated [4]. The exact masses of FB1, pHFB1a, pHFB1b, HFB1 and ^13^C_34_-FB1were also added to the processing method.

**Figure 4:**
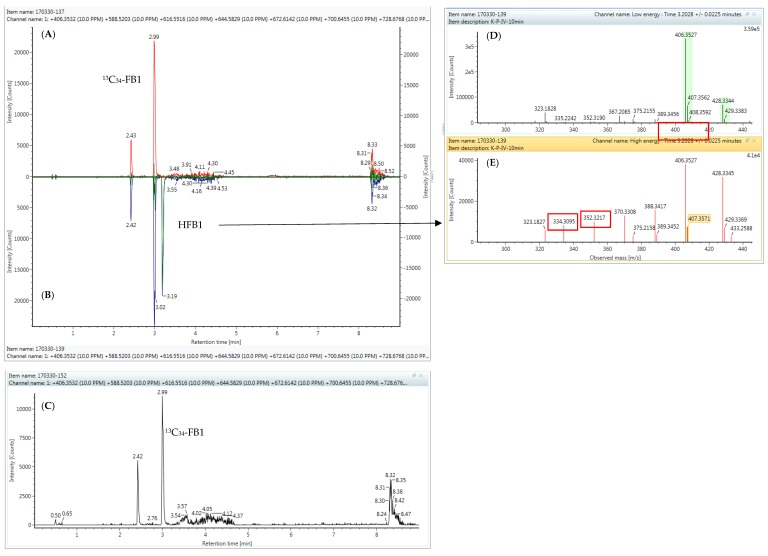
UPLC high-resolution extracted mass chromatograms of plasma samples that were taken from a broiler chicken that received one single intravenous (IV) or oral (OR) dose of 1.25 mg HFB1/kg BW at the following time points: (**A**) prior to IV administration, HFB1 concentration: not detected; (**B**) 10 min after IV administration, HFB1 concentration: 46.0 ng/mL; (**C**) 10 min after OR administration, HFB1 concentration: not detected; the following mass-to-charge (m/z) values, corresponding with the theoretical exact mass of the protonated molecular ions [M-H]^+^, were extracted from the total ion chromatogram: FB1: 722.3963; ^13^C_34_-FB1: 756.5104; pHFB1a+b: 564.3748 and all the m/z values mentioned in Table 2.; (**D**) low energy spectrum of the peak at Tr = 3.19 min in panel B, showing the [M-H]^+^ ion of HFB1 (observed accurate mass at m/z = 406.3527, mass error: 0.0 mDa or 0.1 ppm); (**E**) high energy spectrum of the same peak, showing fragment ions of HFB1 at m/z = 334.3095 and 352.3217.

In Figure 4, extracted mass chromatograms are shown of plasma samples that were taken from a broiler chicken prior to (panel A) and 10 min after the intravenous (IV, panel B) or oral (OR, panel C) administration of HFB1 at a dose of 1.25 mg/kg BW. As can be seen, ^13^C_34_-FB1was detected in all samples. HFB1 could only be detected in the plasma sample that was taken 10 min after IV administration of HFB1. These results were in accordance with those obtained using the UPLC-MS/MS instrument, indicating the successful transfer of the method to the UPLC-HR-MS instrument.

No N-acyl-metabolites of HFB1 could be determined in the chicken plasma samples, as is demonstrated in Figure 4. This could possibly be attributed to the fact that NAHFB1-metabolite concentrations are very low in chicken plasma and fall below the detection capability of the current UPLC-HR-MS method. Further experiments are needed for clarification.

### 2.5. Method Validation

#### 2.5.1. Linearity

All calibration curves were constructed using matrix-matched calibrator samples in order to compensate for matrix effects. Linear calibration curves covering a concentration range of 1–500 ng/mL (FB1 and FB2), 0.86–860 ng/mL (pHFB1a), 0.72–1430 ng/mL (pHFB1b) and 2.5–2500 ng/mL (HFB1) were obtained for all analytes (see Table 3).The correlation coefficients (r) and goodness-of-fit coefficients (gof) were determined and met the acceptance criteria and were ≥0.99 and ≤20%, respectively [23,24]. The residuals plots of the different unweighted calibration curves indicated that the data were heteroscedastic (results not shown), which meant that the absolute error varied with sample concentration. Therefore, the best weighting factor was determined [25]. Per analyte and weighting factor, the sum of goodness-of-fit coefficients (gof) was determined and the results ranged between 178.9–1223.7% (unweighted, 1/x^0^), 19.6–32.8% (weighting factor 1/x^1^) and 12.9–18.7% (weighting factor 1/x^2^). This indicated that a calibration model using a weighting factor of 1/x^2^ was the most appropriate for all analytes. As a consequence, improvements of accuracy were observed particularly at the lower end of the calibration range, resulting in lower limits of quantification [25].

The calibration curves fell in the same range or higher as those reported by Devreese et al. (1–1000 ng/mL for FB1 in pig plasma) [15], Gazotti et al. (5–100 ng/mL for FB1, FB2 and hydrolysed metabolites in pig liver) [7], Han et al. (0.5–60 ng/mL for FB1, FB2 and FB3 in Chinese medicines) [16] or Heinl et al. (10–2000 ng/mL for FB1 and HFB1 in culture medium) [9].

The method of internal standardization was applied in order to compensate for analyte losses during sample preparation and for matrix effects during UPLC-MS/MS analysis. In the presented method, an isotope-labelled internal standard (^13^C_34_-FB1) was chosen, because the structural, physical and chemical properties of such a compound are very similar to FB1. Since ^13^C-labelled standards for pHFB1 and HFB1 are not yet commercially available, ^13^C_34_-FB1 was also used as internal standard for those components. Although this was in theory not optimal, it has been shown during the method validation experiments that the reliable determination of pHFB1a+b and HFB1 was not impaired.

#### 2.5.2. Accuracy and Precision

The within-run accuracy and precision were tested at three different concentration levels (i.e., low, medium and high). The acceptability ranges were met for all compounds at the specified levels according to VICH GL49 [26]. The between-run precision and accuracy was tested at the same concentration levels by the analysis of quality control samples and the results fell also within the ranges specified (see Table 4) [26].

#### 2.5.3. LOQ and LOD

LOQ values in plasma ranged between 0.72 and 2.50 ng/mL, depending on the component. The LOQ was set as the lowest concentration of the calibration curve that could be determined with an accuracy that fell within the acceptability ranges at the specified level.

The LOD values were theoretical and calculated, based on the mean signal-to-noise (S/N) ratios of the LOQ samples. Values corresponding to a theoretical S/N ratio of 3 were set as the LOD and ranged between 0.03 and 0.17 ng/mL. These LOQ and LOD values in plasma were in the same range as reported by Devreese et al. for FB1 in pig plasma [15] and by other authors in matrices of various origin [2,7,12,16]. The LOQ values were low enough to allow accurate quantification of the analytes of interest in plasma samples that were taken from broiler chickens up to 360 min after IV or oral administration of HFB1 (dose: 1.25 mg/kg BW).

#### 2.5.4. Carry-Over

According to Gazotti et al., care must be taken to prevent carry-over problems in the chromatographic system [7]. In the current procedure carry-over was negligible (<0.35%), which can be attributed to the flow-through-needle (FTN)-design of the Acquity H-Class system.

#### 2.5.5. Matrix Effects and Process Efficiency

UPLC-MS/MS is known for its specificity and selectivity, but it has been shown that co-eluting matrix components may affect the ionization efficiency [21,27]. This phenomenon can be reduced by optimizing sample clean-up and chromatographic separation. In the present method the influences of matrix effects have been further minimized by preparing matrix-matched calibrator samples and by the use of an isotope-labelled internal standard.

The influence of the matrix on M_E_ has been investigated quantitatively for FB1, FB2, pHFB1a, pHFB1b and HFB1 [27]. As can be seen from Figure 2B, a moderate signal enhancement (M_E_ : 112.5–127.1%) on the UPLC-MS/MS instrument could be observed for all analytes of interest in chicken plasma if the sample preparation procedure consisted of deproteinization in combination with PL removal using Ostro^TM^ 96-well plates. Using LLE at pH 2 or pH 7, a signal suppression was observed (M_E_ at pH2: 71.2–118.9%; M_E_ at pH 7: 68.3–82.1%).

By combining R_E_ and M_E_, the efficiency (P_E_) of the whole analytical procedure could be evaluated. From the results in Figure 2C, it is clear that the procedure using the Ostro^TM^ 96-well plates is superior compared to LLE at pH 2 or pH 7.

### 2.6. Analysis of Real Samples

**Figure 5:**
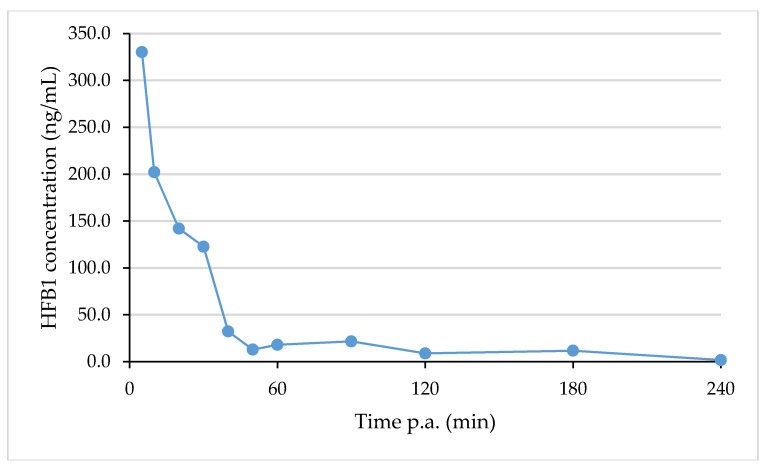
Plasma concentration versus time curve for HFB1 after intravenous administration of HFB1 (dose: 1.25 mg/kg BW) to a 21-day old broiler chicken that received previously a fumonisin-free diet.

To demonstrate the applicability of the developed UPLC-MS/MS method, biological samples were analysed that were taken as a part of a two-way cross-over TK study with HFB1 in chickens that received either a control diet or a FBs contaminated diet, respectively. Blood samples were drawn before and at different time points after the IV and oral administration of a single dose of 1.25 mg HFB1/kg BW. The results of the analysis of HFB1 in plasma samples that were taken from one chicken that received a control diet and an IV dose of HFB1 are shown in Figure 5.

As can be seen from Figure 5, HFB1 concentrations above the LOQ level (2.5 ng/mL) could be detected up to 180 min after IV administration. HFB1 concentrations after oral administration were much lower and concentrations above the LOQ level could only be determined in some samples.

The above results indicate the applicability of the developed method, not only for the determination of the TK of HFB1 in broiler chickens, but also for future TK studies with FBs, pHFBs and HFBs in different animal species. More details concerning the animal experiment and the TK parameters of HFB1 will be presented in a forthcoming article by Antonissen et al. [28].

## 3. Conclusions

This study describes the development and in-house validation of a sensitive and specific UPLC-MS/MS method for the quantitative determination of FB1 and its hydrolysed metabolites (pHFB1a, pHFB1b, HFB1) in plasma of broiler chickens. Special emphasis was made on a high-throughput sample analysis, which is needed to process a large number of samples for TK studies. Therefore, the sample preparation consisted of a combined protein precipitation and PL removal for plasma using an Oasis^®^ Ostro^TM^ 96-well plate. Furthermore, chromatographic separation was achieved within a short run-time of 10 min. The method was successfully validated for all analytes of interest in chicken plasma according to international regulations and literature [23,24,25,26,27,29,30] and detailed results (linearity, precision, accuracy, LOQ, LOD, specificity) were presented.

Incurred plasma samples that were taken during a TK study with HFB1 from broiler chickens that received previously a control diet or a fumonisin-contaminated diet, were quantitatively analysed using the described UPLC-MS/MS method. HFB1 was quantitated both after IV and OR administration.

To investigate the presence of N-acyl-metabolites of HFB1 in chicken plasma, the same samples were analysed using UPLC-HR-MS. No NAHFB1-metabolites could be determined using the current method.

In conclusion, the presented method can be applied in the field of TK analysis, not only for HFB1, but also for future TK studies with FBs, pHFBs and HFBs in different animal species.

## 4. Materials and Methods

### 4.1. Chemicals and Reagents

The standards of FB1, FB2 and FB3 were obtained from Fermentek Ltd (Jerusalem, Israel) and stored at 2–8 °C. The internal standard (IS), ^13^C_34_-FB1 was purchased as a 25 µg/mL solution in acetonitrile/water (50/50, *v/v*) from Biopure (Tulln, Austria) and stored at 2–8 °C. A standard mixture containing FB1 (2.9 µg/mL), HFB1 (5.0 µg/mL), pHFB1a (8.3 µg/mL) and pHFB1b (14.5 µg/mL) was prepared by Biomin according to the procedure described by Hahn et al. [2]. This standard mixture was stored at ≤−15 °C.

The solvents and reagents that were used for the preparation of the UPLC mobile phase (water, ACN, FA, NH_4_FA) were of UPLC-MS grade and were obtained from Biosolve (Valkenswaard, The Netherlands). All other solvents and reagents were of HPLC grade (water, ACN, diethylether, EtOAc; Filterservice, Eupen, Belgium) or analytical grade (FA, hydrochloric acid; VWR, Leuven, Belgium).

Oasis^®^ Ostro^TM^ protein precipitation & phospholipid removal 96-well plates (25 mg) were obtained from Waters (Zellik, Belgium).

### 4.2. Preparation of Standard Solutions

Stock solutions of FB1, FB2 and FB3 (1 mg/mL) were prepared in water/ACN (50/50, *v/v*) and stored at 2–8 °C. Working solutions at concentrations of 10 µg/mL, 1 µg/mL and 0.1 µg/mL were prepared by appropriate dilution of the stock solution in water/ACN (50/50, *v/v*). The standard mixture solution containing FB1, HFB1, pHFB1a and pHFB1b was appropriately diluted in water/ACN (50/50, *v/v*) to obtain working solutions for the preparation of calibrator and quality control (QC) samples. For the IS, a working solution of 1 µg/mL was prepared in water/ACN (50/50, *v/v*). All working solutions were stored at 2–8 °C.

### 4.3. Biological Samples

Blank plasma samples. For the preparation of matrix-matched calibrator and QC samples, blank plasma samples were obtained from broiler chickens that received no FBs. Blank samples were stored at ≤−15 °C until the moment of analysis.

Incurred plasma samples. In order to evaluate the applicability of the developed method, incurred plasma samples from broiler chickens were analysed.

Chicken plasma samples were obtained after the IV and OR administration of HFB1 (dose: 1.25 mg/kg BW), to 21-day-old chickens that received previously a control (i.e., fumonisin-free) diet (*n* = 8) or a fumonisin-contaminated diet for two weeks (*n* = 8, i.e., between day 8 and day 21, fumonisin contamination level of the feed: 10.8 mg/kg FB1, 3.3 mg/kg FB2 and 1.5 mg/kg FB3). A two-way cross-over design was applied and with a wash-out and recovery period of two days between both administrations. Blood samples were taken from the leg vein at 0 min (before administration) and at 5′, 10′, 20′, 30′, 40′, 50′, 1 h, 1.5 h, 2 h, 3 h, 4 h, 6 h, and 9 h post administration (p.a.). After sampling, all blood samples were centrifuged at 2851× *g* for 10 min (4 °C) and plasma was stored at ≤−15 °C until the moment of analysis. The animal experiment was approved by the Ethical Committee of the Faculty of Veterinary Medicine and Bioscience Engineering of Ghent University (EC 2015/10, approval date: 9 March 2015).

The results of the analysis of the plasma samples of one broiler chicken are presented in Section 2.6. Detailed results concerning the plasma concentration versus time profiles and the toxicokinetic parameters of HFB1 after IV and OR administration will be presented in a forthcoming article [28].

### 4.4. Sample Pre-Treatment

#### 4.4.1. Final Procedure

To 100 µL of plasma were added 12.5 µL of the IS working solution (1.0 µg/mL), followed by vortex mixing and loading onto the Ostro^TM^ 96-well plate. Thereafter, 300 µL of 1% FA in ACN were added and the sample was aspirated three times to enhance protein precipitation. The sample was passed through the 96-well plate by the application of a vacuum (67.7 kPa) for 10 min. A 2.5-µL aliquot was injected onto the UPLC-MS/MS or UPLC-HR-MS instrument. 

#### 4.4.2. Liquid–Liquid Extraction Procedure

To 100 µL of plasma were added 12.5 µL of the IS working solution (1.0 µg/mL), followed by vortex mixing and the addition of 3 mL of ether or EtOAc. The sample was extracted in acidic (pH = 2) or neutral (pH = 7) medium for 20 min on a horizontal rotary shaker, followed by a centrifugation step of 10 min at 3500 rpm. Thereafter, the organic phase was transferred to another tube and evaporated until dryness under a gentle stream of nitrogen at a temperature of ±45 °C. The dry residue was reconstituted in 250 µL of a water/ACN (50/50, *v/v*) mixture, vortex mixed and transferred to an autosampler vial. A 2.5-µL aliquot was injected onto the UPLC-MS/MS instrument.

### 4.5. UPLC-MS/MS Analysis for Quantification

The UPLC system consisted of an Acquity UPLC H-Class Quaternary Solvent Manager and Flow-Through-Needle Sample Manager with temperature controlled tray and column oven from Waters (Zellik, Belgium). Chromatographic separation was achieved on an Acquity UPLC HSS T3 column (100 mm × 2.1 mm i.d., dp: 1.8 µm) in combination with an Acquity HSS T3 1.8 μm Vanguard pre-column, both from Waters. 

The mobile phase A consisted of 0.3% FA and 10 mM NH_4_FA in water, while the mobile phase B was acetonitrile. A gradient elution was performed: 0–0.5 min (90% A, 10% B), 5.5 min (linear gradient to 90% B), 5.5–7.5 min (10% A, 90% B), 7.7 min (linear gradient to 90% A), 7.7–10.0 min (90% A, 10% B). The flow-rate was 0.4 mL/min.

The temperatures of the column oven and autosampler tray were set at 40 °C and 8 °C, respectively.

The UPLC column effluent was interfaced to a Xevo TQ-S^®^ MS/MS system, equipped with an ESI probe operating in the positive mode (all from Waters). A divert valve was used and the UPLC effluent was directed to the mass spectrometer from 2.5 to 4.5 min.

Instrument parameters were optimised by direct infusion of working solutions of 1.0 µg/mL of FB1, FB2, FB3 and the IS and of a diluted standard mixture solution, containing FB1, HFB1, pHFB1a and pHFB1b at a concentration of 0.50, 0.86, 1.43 and 2.50 µg/mL, respectively, at a flow-rate of 10 µL/min and in combination with the mobile phase (50% A, 50% B, flow-rate: 200 µL/min).

The following parameters were used: capillary voltage: 3.0 kV, cone: 40 V, source offset: 60 V, desolvation temperature: 600 °C, desolvation gas: 1000 L/h, cone gas: 150 L/h, nebuliser pressure: 7.0 bar, LM resolution 1 and 2: 2.80 and 2.77, respectively, HM resolution 1 and 2: 15.00, respectively, ion energy 1 and 2: 0.2 and 0.8, respectively, collision gas flow: 0.15 mL/min.

MS/MS acquisition was performed in the MRM mode. The MRM transitions that were monitored for all analytes are shown in Table 1.

### 4.6. UPLC-HR-MS Analysis for Identification

An Acquity I-Class UPLC coupled to a Synapt G2-S*i* HDMS instrument (Waters, Zellik, Belgium) was used to identify potential phase-II metabolites and N-acyl metabolites of HFB1 in incurred chicken plasma samples. The chromatographic conditions were the same as described above. HR-MS instrument parameters were optimized by syringe infusion of a standard mixture solution of FB1, pHFB1a, pHFB1b and HFB1. The following HR-MS parameters were used: capillary voltage, 2.70 kV; sampling cone voltage, 30.00 V; source offset, 80.00 V; source temperature, 150 °C; desolvation temperature, 550 °C; cone gas flow, 50 L/h; desolvation gas flow, 800 L/h; nebuliser gas flow, 6.50 bar; lock spray capillary voltage, 2.00 kV. HR-MS acquisition was performed from 0.2–9.0 min in the positive ESI resolution mode using the MS^E^ continuum scan function. Time-of-flight (TOF) MS settings were as follows: low mass, 50 Da; high mass, 1000 Da; scan time, 0.15 s; interscan time, 0.1 s; data format, continuum. The lock mass solution consisted of leucine encephalin (200 pg/µL). The lockspray was acquired during HR-MS acquisition, but no correction was applied. The lock spray settings were as follows: scan time, 0.15 s; interval, 30 s; scans to average, 3; mass window, 0.5 Da. Data processing and lock mass correction (m/z 556.276575) was performed using the Unify 1.8 software (Waters). Identification of analytes was based on retention time (target T_R_ tolerance: 0.1 min) and mass (target mass tolerance: 10 ppm). The search for phase-II metabolites of HFB1 was performed using a pathway profiling approach. The following transformations were added to the method: glucuronide conjugation and sulphate conjugation, based on the suggestion by Hopmans et al. [31]. The search for N-acyl metabolites of HFB1 was based on Harrer et al., 2015 and Seiferlein et al.*,* 2007 and was performed using a targeted approach [4,5]. Therefore, the chemical formulas of potential N-acyl (NA) metabolites of HFB1 (ranging from C12-HFB1 to C26-HFB1 and C24:1-HFB1, C26:1-HFB1) were added to the accurate mass–MSe screening method (see Table 2).

### 4.7. Method Validation

The developed LC-MS/MS method was in-house validated for FB1, FB2, pHFB1a, pHFB1b and HFB1 based on the protocol described by De Baere et al. [23], using spiked blank plasma samples obtained from healthy, untreated chickens. The method was not validated for FB3, since this component was of less importance (cfr. Concentration in contaminated feed) and it was expected that eventual FB3 levels in plasma would be very low.

Linearity, accuracy, precision, limit of quantification (LOQ), limit of detection (LOD) and carry-over were determined in compliance with the recommendations and guidelines defined by the European Community and with criteria described in the literature [23,24,25,26,27,29,30].

#### 4.7.1. Calibration Curves

Matrix-matched calibration curves were prepared in 100 µL blank chicken plasma (concentration range: FB1: 1–500 ng/mL, FB2: 1–500 ng/mL, pHFB1a: 0.86–860 ng/mL, pHFB1b: 0.72–1430 ng/mL, HFB1: 2.5–2500 ng/mL). Three individual calibration curves were prepared, i.e., one on three different analysis days. The correlation coefficients (r) and gof were calculated and limits were set at ≥0.99 and ≤20%, respectively [23,24,30]. The gof was determined according to Knecht and Stork [24] using the following formula:

(1) $$g=\sqrt{\Sigma^{\text{​}}{\left(\%\;\mathrm{deviation}\right)}^{2}/(n-1)},\;{\mathrm{with}\;\%\;\mathrm{deviation}=x}_{\mathrm{calculated}\;\mathrm{conc}}-\;x_{\mathrm{nominal}\;\mathrm{value}}/x_{\mathrm{nonimal}\;\mathrm{value}}\times 100.$$

The choice of the most appropriate weighting factor (1/x^0^, 1/x^1^, 1/x^2^) was performed, based on VICH GL49 and Almeida et al. [25,26]. The following procedure was applied: three weighting factors were applied to each calibration curve and the gof were calculated. Per weighting factor, the gof of the three individual curves were summed; the weighting factor that gave the smallest sum of gof was considered to be the most appropriate model.

#### 4.7.2. Accuracy and Precision

Within-run accuracy and precision (repeatability) were determined by analysing six blank samples that were spiked at a low medium and high concentration level in the same run. The between-run accuracy and precision (reproducibility) were determined by analysing two blank samples spiked at the same concentration levels on three different days (*n* = 6). An overview of the concentration levels per component is given in Table 3. The acceptance criteria for accuracy were: −50% to +20%, 40% to +20%, −30% to +10% and −20% to +10% for concentration levels <1 ng/mL, ≥1 to <10 ng/mL, ≥10 to <100 ng/mL and ≥100 ng/mL, respectively. The precision was evaluated by the determination of the relative standard deviation (RSD), which had to be below the RSD_max_ value. For the within-day precision, RSD_max_ is fixed at <30%, <25%, <15% and <10% for concentrations <1 ng/mL, ≥1 to <10 ng/mL, ≥10 to <100 ng/mL and ≥100 ng/mL, respectively [26]. For between-run precision, the RSD had to be below the RSD_max_ value calculated by the Horwitz equation [26]. These criteria are shown in Table 4.

#### 4.7.3. LOQ and LOD

The LOQ was the lowest concentration of the analyte for which the method was validated with an accuracy that fell within the recommended range. The LOQ was also established as the lowest point of the calibration curve. The LOQ was determined by analysing six samples spiked at a concentration level of 1.0 ng/mL for FB1 and FB2, 0.86 ng/mL for pHFB1a, 0.72 ng/mL for pHFB1b and 2.5 ng/mL for HFB1, on the same day. In addition, the S/N ratios of all analytes in the LOQ samples were determined and the mean S/N ratios were calculated.

The LOD was defined as the concentration that corresponded with a theoretical S/N ratio of 3. The LOD values were calculated using the mean S/N of the analytes spiked in blank plasma at the LOQ level.

#### 4.7.4. Carry-Over

The absence of carry-over was verified by analysing the reconstitution solvent injected after the highest calibration sample. If a peak was observed in the elution zone of an analyte or the IS, it had to be below the LOD.

#### 4.7.5. Extraction Recovery, Matrix Effects and Process Efficiency

Extraction recovery (R_E_), matrix effects (M_E_) and process efficiency (P_E_) were quantitatively assessed by preparing three sets of samples: set A consisted of standard solutions containing the analytes of interest at a concentration of 250 ng/mL (FB1 and FB2), 430 ng/mL (pHFB1a), 715 ng/mL (pHFB1b) and 1250 ng/mL (HFB1); the other sets consisted of matrix-matched samples that were prepared by spiking blank matrix after (set B) and before (set C) extraction at the same concentration levels as the set-A samples. All experiments were performed in triplicate. The R_E_, M_E_ and P_E_ were determined by dividing the peak areas of FB1, FB2, pHFB1a, pHFB1b and HFB1 in the respective samples, i.e., R_E_ = C/B × 100, M_E_ = B/A × 100 and P_E_ = C/A × 100.

## Figures and Tables

**Table:** 

Analyte	R1	R2	R3	R4	R5
FB1	TCA	TCA	OH	OH	H
FB2	TCA	TCA	H	OH	H
FB3	TCA	TCA	OH	H	H
pHFB1a	TCA	H	OH	OH	H
pHFB1b	H	TCA	OH	OH	H
HFB1	H	H	OH	OH	H
NAHFB1	H	H	OH	OH	NA

**Table 1:** MRM transitions and MS/MS parameters for the analytes under investigation (UPLC-MS/MS analysis).

Analyte	Chemical Formula	MM ^a^ (g/mol)	Precursor Ion (m/z) ^b^	Product Ions (m/z)	CE ^c^ (eV)	Cone (V)	Retention Time (min)
FB1	C_34_H_59_NO_15_	721.3885	722.2	334.2	33	40	3.29
				352.2 ^d^	33	40	
pHFB1a	C_28_H_53_NO_10_	563.3669	564.3	334.3	27	40	3.42
				352.3 ^d^	27	40	
pHFB1b	C_28_H_53_NO_10_	563.3669	564.3	334.3	27	40	3.56
				352.3 ^d^	27	40	
HFB1	C_22_H_47_NO_5_	405.3454	406.3	334.2 ^d^	25	40	3.80
				352.3	25	40	
FB2	C_34_H_59_NO_14_	705.3936	706.0	336.2 ^d^	35	40	3.84
				354.2	35	40	
FB3	C_34_H_59_NO_14_	705.3936	706.0	336.2 ^d^	35	40	3.59
				354.2	35	40	
^13^C_34_-FB1	^13^C_34_H_59_NO_15_	755.5025	756.4	356.4	35	40	3.28
				374.4 ^d^	35	40	

^a^ MM = monoisotopic mass, ^b^ m/z = mass to charge ratio, ^c^ CE = collision energy, ^d^ ion used for quantification.

**Table 2:** Overview of chemical formulas and theoretical exact masses of the N-acyl derivatives of HFB1.

Component	Chemical Formula	Chemical Formula N-acyl Chain	Theoretical Exact Mass (g/mol)	Theoretical Exact Mass Protonated Molecular Ion [M-H]^+^ (m/z) ^a^
HFB1	C_22_H_47_O_5_N		405.3454	406.3532
C_12_-HFB1	C_34_H_69_O_6_N	+C_12_H_22_O	587.5125	588.5203
C_14_-HFB1	C_36_H_73_O_6_N	+C_14_H_26_O	615.5438	616.5516
C_16_-HFB1	C_38_H_77_O_6_N	+C_16_H_30_O	643.5751	644.5829
C_18_-HFB1	C_40_H_81_O_6_N	+C_18_H_34_O	671.6064	672.6142
C_20_-HFB1	C_42_H_85_O_6_N	+C_20_H_38_O	699.6377	700.6455
C_22_-HFB1	C_44_H_89_O_6_N	+C_22_H_42_O	727.6690	728.6768
C_24_-HFB1	C_46_H_93_O_6_N	+C_24_H_46_O	755.7003	756.7081
C_26_-HFB1	C_48_H_97_O_6_N	+C_26_H_50_O	783.7316	784.7394
C_24:1_-HFB1	C_46_H_91_O_6_N	+C_24_H_44_O	753.6846	754.6925
C_26:1_-HFB1	C_48_H_95_O_6_N	+C_26_H_48_O	781.7159	782.7238

^a^ m/z = mass to charge ratio.

**Table 3:** Results of the evaluation of linearity (slope (a), intercept (b), goodness-of-fit coefficient (gof), correlation coefficient (r)), limit of quantification (LOQ), calculated limit of detection (LOD) for fumonisin B1 (FB1), partially hydrolysed FB1 (pHFB1a and pHFB1b) and hydrolysed FB1 (HFB1) in broiler chicken plasma.

Component	Calibration Range (ng/mL)	Spike Levels (ng/mL)	A ^a^	b ^a^	gof ^a^ (%)	r ^a^	LOQ (ng/mL)	LOD ^c^ (ng/mL)
FB1	1–500	1, 2.5, 5, 10, 25, 50, 100, 250, 500	0.00869 ± 0.00026	−0.00207 ± 0.00722	5.25 ± 1.18	0.99825 ± 0.00085	1.0 (S/N ^b^ = 20.1)	0.15
FB2	1–500	1, 2.5, 5, 10, 25, 50, 100, 250, 500	0.013669 ± 0.00152	−0.00506 ± 0.00395	6.22 ± 1.14	0.99757 ± 0.00106	1.0 (S/N = 33.3)	0.09
pHFB1a	0.86–860	0.86, 1.72, 4.3, 8.6, 17.2, 43, 86, 172, 430, 860	0.06929 ± 0.00767	−0.01107 ± 0.00632	5.72 ± 1.82	0.99798 ± 0.00105	0.86 (S/N = 69.5)	0.04
pHFB1b	0.72–1430	0.72, 1.43, 2.86, 7.15, 14.3, 28.6, 71.5, 143, 286, 715, 1430	0.05665 ± 0.00702	−0.01758 ± 0.00927	4.30 ± 3.78	0.99831 ± 0.00161	0.72 (S/N = 70.3)	0.03
HFB1	2.5–2500	2.5, 5, 12.5, 25, 50, 125, 250, 500, 1250, 2500	0.01240 ± 0.00285	−0.00280 ± 0.00362	5.99 ± 0.85	0.99795 ± 0.00058	2.5 (S/N = 45.4)	0.17

Note: ^a^ Mean results (*n* = 3) ± standard deviation are shown; ^b^ S/N: mean signal-to-noise ratio of the analytes in the LOQ samples (*n* = 6); ^c^ LOD : the limit of detection is the theoretical concentration that corresponds with a S/N ratio of 3 and was calculated using the mean S/N ratio of the analytes in the LOQ samples.

**Table 4:** Results of the within-run and between-run precision and accuracy evaluation for the analysis of fumonisin B1 (FB1), fumonisin B2 (FB2), partially hydrolysed FB1 (pHFB1a and pHFB1b) and hydrolysed FB1 (HFB1) in chicken plasma.

Component	Theoretical Concentration (ng/mL)	Mean Concentration ± SD (ng/mL)	Precision, RSD (%)	Accuracy (%)
FB1	1.0 ^a^	1.01 ± 0.11	10.7	1.2
	2.5 ^a^	2.4 ± 0.39	16.4	−3.5
	2.5 ^b^	2.4 ± 0.58	24.7	−5.5
	25.0 ^a^	25.2 ± 1.82	7.2	0.7
	25.0 ^b^	23.6 ± 1.66	7.1	−5.8
	250.0 ^a^	243.8 ± 10.19	4.2	−2.5
	250.0 ^b^	251.0 ± 8.20	3.3	0.4
FB2	1.0 ^a^	0.77 ± 0.15	19.0	−23.0
	2.5 ^a^	2.2 ± 0.40	18.0	−12.2
	2.5 ^b^	2.3 ± 0.21	9.1	−9.9
	25.0 ^a^	24.0 ± 1.11	4.6	−4.0
	25.0 ^b^	24.2 ± 2.80	11.6	−3.3
	250.0 ^a^	246.5 ± 9.75	4.0	−1.4
	250.0 ^b^	253.1 ± 16.91	6.7	1.3
pHFB1a	0.86 ^a^	1.02 ± 0.05	5.3	19.1
	4.3 ^a^	4.1 ± 0.28	6.9	−4.7
	4.3 ^b^	4.0 ± 0.33	8.2	−7.6
	43.0 ^a^	34.4 ± 3.49	10.2	−20.0
	43.0 ^b^	39.1 ± 1.97	5.0	−9.1
	430.0 ^a^	387.8 ± 38.24	9.9	−9.8
	430.0 ^b^	425.7 ± 16.00	3.8	−1.0
pHFB1b	0.72 ^a^	0.77 ± 0.13	16.4	7.0
	7.2 ^a^	6.3 ± 0.32	5.0	−11.7
	7.2 ^b^	6.5 ± 0.31	4.7	−8.6
	71.5 ^a^	57.6 ± 5.19	9.0	−19.5
	71.5 ^b^	63.7 ± 1.64	2.6	−10.9
	715.0 ^a^	682.4 ± 51.44	7.5	−4.6
	715.0 ^b^	714.9 ± 29.66	4.1	0.0
HFB1	2.5 ^a^	2.4 ± 0.40	16.6	−3.1
	12.5 ^a^	12.5 ± 0.81	6.4	−0.1
	12.5 ^b^	11.3 ± 1.59	14.0	−9.7
	125.0 ^a^	115.9 ± 7.15	6.2	−7.3
	125.0 ^b^	122.2 ± 10.9	8.9	−2.3
	1250.0 ^a^	1225.9 ± 57.79	4.7	−1.9
	1250.0 ^b^	1253.6 ± 97.29	7.8	0.3

^a^ Within-run accuracy and precision (*n* = 6); ^b^ Between-run accuracy and precision (*n* = 6); SD: standard deviation; RSD: relative standard deviation; Acceptance criteria [26]: accuracy: <1 ng/mL: −50% to +20%, ≥1 to <10 ng/mL: −40% to +20%, ≥10 to <100 ng/mL: −30% to +10%, ≥100 ng/mL: −20% to +10%; within-run precision (RSD_max_): <1 ng/mL: 30.0%, ≥1 to <10 ng/mL : 25.0%, ≥10 to <100 ng/mL: 15.0%, ≥100 ng/mL: 10.0%, between-run precision, as determined by the Horwitz equation, RSD_max_ = 2^(1^^−^^0.5logC)^, where C = concentration expressed as a decimal fraction (e.g., 1 ng/mL is entered as 10^−^^9^): <1 ng/mL: 45.0%, ≥1 to <10 ng/mL : 32.0%, ≥10 to <100 ng/mL: 23.0%, ≥100 ng/mL: 16.0%.
